# Alteration of Gene Expression Signatures of Cortical Differentiation and Wound Response in Lethal Clear Cell Renal Cell Carcinomas

**DOI:** 10.1371/journal.pone.0006039

**Published:** 2009-06-25

**Authors:** Hongjuan Zhao, Zongming Ma, Robert Tibshirani, John P. T. Higgins, Börje Ljungberg, James D. Brooks

**Affiliations:** 1 Department of Urology, Stanford University, Stanford, California, United States of America; 2 Department of Statistics, Stanford University, Stanford, California, United States of America; 3 Department of Pathology, Stanford University, Stanford, California, United States of America; 4 Department of Urology, Umeå University, Umeå, Sweden; Baylor College of Medicine, United States of America

## Abstract

Clear cell renal cell carcinoma (ccRCC) is the most common malignancy of the adult kidney and displays heterogeneity in clinical outcomes. Through comprehensive gene expression profiling, we have identified previously a set of transcripts that predict survival following nephrectomy independent of tumor stage, grade, and performance status. These transcripts, designated as the SPC (supervised principal components) gene set, show no apparent biological or genetic features that provide insight into renal carcinogenesis or tumor progression. We explored the relationship of this gene list to a set of genes expressed in different anatomical segments of the normal kidney including the cortex (cortex gene set) and the glomerulus (glomerulus gene set), and a gene set expressed after serum stimulation of quiescent fibroblasts (the core serum response or CSR gene set). Interestingly, the normal cortex, glomerulus (part of the normal renal cortex), and CSR gene sets captured more than 1/5 of the genes in the highly prognostic SPC gene set. Based on gene expression patterns alone, the SPC gene set could be used to sort samples from normal adult kidneys by the anatomical regions from which they were dissected. Tumors whose gene expression profiles most resembled the normal renal cortex or glomerulus showed better survival than those that did not, and those with expression features more similar to CSR showed poorer survival. While the cortex, glomerulus, and CSR signatures predicted survival independent of traditional clinical parameters, they were not independent of the SPC gene list. Our findings suggest that critical biological features of lethal ccRCC include loss of normal cortical differentiation and activation of programs associated with wound healing.

## Introduction

In the United States, it is estimated that approximately 54,390 renal cell carcinomas (RCCs) will be diagnosed in 2008, and 13,010 patients will die of their disease [1]. Clear cell renal cell carcinoma (ccRCC) accounts for approximately 75% of all RCCs, the most common malignancy of the adult kidney, and the majority of kidney cancer mortality [2]. Surgery (nephrectomy) can cure 60%–70% of patients with localized disease and prolong survival in patients with metastatic disease [3]. While stage, grade and patient performance status are the most important predictors of treatment failure they are only modestly successful prognosticators, likely due to underlying molecular heterogeneity that influences biological behavior [4]. Targeted therapies have recently emerged that have dramatically altered management of ccRCC, however, less than half of tumors respond, again likely reflecting intrinsic differences in the biology of these tumors. Currently, prognosis after surgery can only be crudely predicted using traditional clinical outcome measures, and new prognostic factors and predictive strategies are urgently needed [5].

We and others have used gene expression profiling to identify gene signatures predictive for outcome in ccRCC. Using a semisupervised learning algorithm (supervised principal components (SPC) analysis), we identified 259 genes represented by 340 transcripts whose expression was associated with survival in a training set of 88 primary ccRCCs [6]. This gene expression-based survival predictor, designated the SPC gene set, was a strong predictor of survival independent of tumor stage, grade, and performance status in an independent validation set of 89 cases [6]. One interesting feature of the SPC gene set was that 95% of the genes show relatively high levels of expression in tumors from patients with prolonged survival after surgery and low expression in those with short survival. A similar pattern of expression was observed by Takahashi et al. in a set of 51 prognostic genes identified in 29 ccRCCs [7]. Moreover, 15 of these 51 prognostic genes were found in the SPC gene set, representing a significant enrichment. However, analysis of the known functions of genes in the SPC set was unrevealing and showed no enrichment of biological pathways or functional groups [6]. Therefore, while the gene sets identified were powerful predictors of outcome, they yielded few insights into the biological features driving an aggressive phenotype in ccRCC.

Understanding prognostic signatures discovered by statistical methods that are agnostic with respect to biology could be facilitated by comparison to other gene expression signatures identified with a predefined hypothesis and linked to particular biological processes. To identify biological threads in the SPC signature that would provide insight into ccRCC tumor biology, we compared this gene list to a set of genes expressed in different anatomical segments of the normal kidney [8] and a gene set expressed after serum stimulation of quiescent fibroblasts [9]. In addition, gene signatures of the normal kidney and serum response signatures were tested for their ability to predict outcome in our dataset of 177 ccRCC patient samples.

## Materials and Methods

### ccRCC gene expression and clinical data sets

The ccRCC gene expression data set was generated using fresh-frozen tissues from 177 patients who underwent radical nephrectomy between 1985 and 2003 in the Department of Urology, Umeå University Hospital (Umeå, Sweden). A detailed description of the cohort was reported previously [6]. Gene expression profiling had been carried out on cDNA microarrays containing over 40,000 clones representing 27,290 unique Unigene clusters. Fluorescence levels of total RNA compared to Universal Human Reference RNA (Strategene, La Jolla, California, USA) were obtained using an Axon GenePix 4000B scanner, with quality control metrics described previously [6]. Fluorescent ratios were normalized by mean-centering genes for each microarray, mean centering each gene across all microarrays and correcting for print run bias as described previously [6]. Missing data was imputed by averaging expression values from the 5 nearest neighbors. The complete microarray dataset is available at http://smd.stanford.edu/cgi-bin/publication/viewPublication.pl?pub_no484. The data also have been deposited in National Center for Biotechnology Information's Gene Expression Omnibus (Accession Number GSE3538). We followed the MIAME (Minimum Information About a Microarray Experiment) guidelines in experimental design, data analyses and annotation.

### Experimentally derived gene expression sets

The SPC gene set includes 259 unique named genes represented by 340 transcripts that are highly prognostic for death due to ccRCC independent of clinical and pathological features. The SPC gene set was identified using supervised principal components analysis as described previously [6]. The normal kidney gene expression data set was generated using dissected renal lobes of five adult human kidneys [8]. The complete microarray dataset containing 34 samples from the inner and outer cortex, inner and outer medulla, papillary tips, and renal pelvis and from glomeruli is available at https://www.med.stanford.edu/jhiggins/Normal_Kidney/index.shtml. The cortex gene set encompasses 190 unique named genes represented by 260 transcripts identified by unsupervised hierarchical clustering analysis [8]. The glomerulus gene set consists of 71 unique named genes represented by 132 transcripts identified using the same method as the cortex gene set [8]. All transcripts in the cortex and glomerulus gene set are expressed at high levels compared to the other anatomical segments of the normal kidney.

The Core Serum Response (CSR) gene set was derived from serum stimulation of synchronized fibroblasts from diverse body sites using the Significance Analysis of Microarrays (SAM) procedure [10]. Transcripts highly correlated with proliferation were removed resulting in 400 unique named genes represented by 573 transcripts as described previously [9]. The CSR captures many transcriptional features normally activated during wound healing.

### Statistical Analysis

#### Hierarchical clustering analysis

Among all genes that were well measured (*n* = 14,814) in the ccRCC dataset, we identified two gene subsets that overlapped with the cortex gene or the CSR gene lists: the first subset consisted of 87 “normal cortex” transcripts and the second included 420 CSR transcripts. We defined well-measured genes as those with a ratio of signal intensity to background noise of more than 1.5 for either the Cy5-labeled ccRCC sample or the Cy3-labeled reference sample, in at least 70% of the samples hybridized. For each gene subset, we performed average-linkage hierarchical clustering which segregated the samples into two major groups based on the first bifurcation of the dendrogram. For the normal cortex gene set, the survival times of the resulting 2 groups were compared using Kaplan-Meier survival analysis and the log-rank test using WinSTAT software. The CSR gene set also segregated tumors into two groups and these were compared similarly.

#### Scaling gene expression signatures

The normalized fluorescence ratios of the 87 normal cortex gene transcripts in the ccRCC dataset were added to yield to a quantitative score reflecting the degree of enrichment of the cortex signature for each sample. Since all of these transcripts were expressed at high levels in the normal renal cortex, the higher the cortex signature value in the kidney tumors, the more the sample resembled the normal renal cortex transcript signature. For each of the 420 CSR related transcripts in the ccRCC dataset, we assigned +1 to each transcript if it was expressed at high levels in fibroblasts after serum stimulation, and −1 if expressed at a low level. The sign corrected, normalized fluorescence ratios for each of the 420 CSR transcripts in the ccRCC dataset were summed to provide a score that reflected the resemblance to the CSR signature for each sample. Higher scores corresponded to enrichment for expression profiles observed in serum-activated fibroblasts.

#### Multivariate proportional-hazards analysis

For both cortex and CSR signatures, we performed multivariate proportional-hazards analysis by using either signature as a continuous variable, together with tumor stage, tumor grade, and patient performance status. We also performed the multivariate analysis with group labeling obtained in hierarchical clustering as a categorical variable. Multivariate proportional-hazards analysis was performed using the R software package (available at http://www.r-project.org).

## Results

### Normal renal cortex, glomerulus and core serum response (CSR), overlap significantly with the SPC gene set

**Figure 1:**
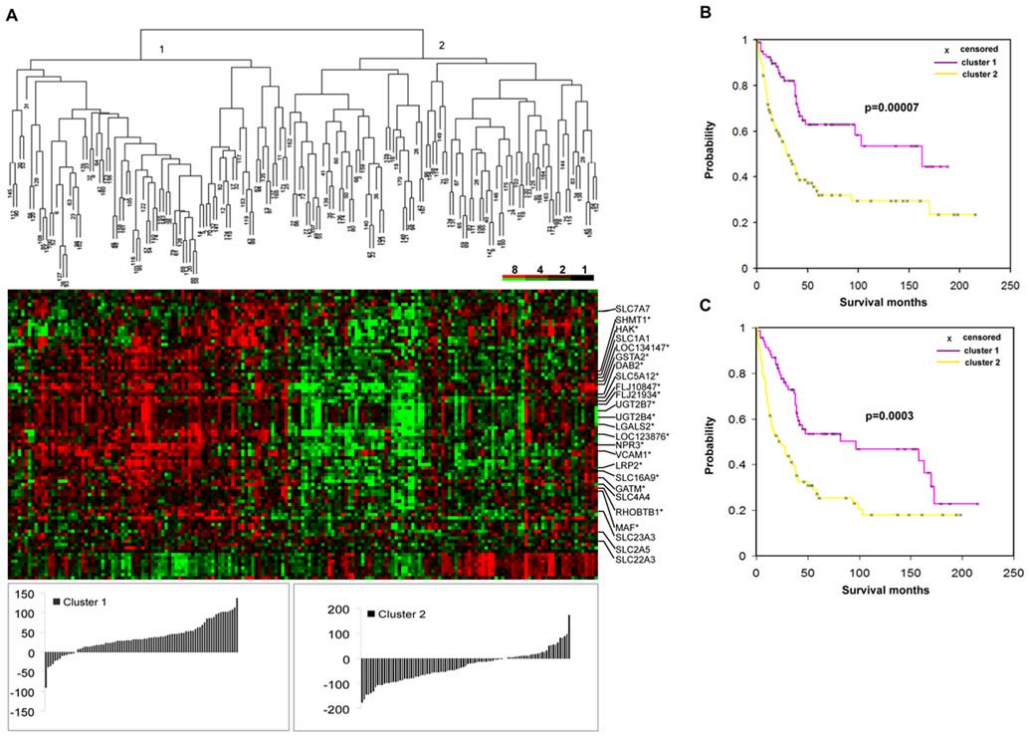
Expression of normal cortex genes predicts survival in ccRCC. (A) Supervised hierarchical clustering analysis of the expression patterns of 67 cortex genes represented by 87 transcripts in 177 ccRCCs. Each row represents a single gene, and each column a patient sample. The degree of color saturation corresponds to the ratio of gene expression in each sample compared to the mean expression across all samples. The distributions of the scaled gene expression scores were shown below the heat map. (B) Kaplan-Meier estimates of disease-specific survival of two main groups of ccRCC patients defined in (A) based on the cortex gene expression. (C) Kaplan-Meier estimates of overall survival of two main groups of ccRCC patients defined in (A) based on the cortex gene expression.

Although the SPC gene set was highly prognostic, analysis of the known functions of each of the 259 genes in this set did not reveal common pathways or biological themes that could provide insights into the biology of lethal ccRCC [6]. To gain insights, we compared the SPC gene set to another gene expression data set identified by profiling different anatomical segments of the normal kidney including the glomeruli, cortex, medulla, papillary tips, and pelvis. The expression patterns of these genes represent the transcriptional programs intrinsic to each of these kidney segments in their normal, fully differentiated state. The cortex signature consists of 190 named unique genes represented by 260 clones, out of which 25 named genes were found in SPC gene list (Figure 1A). This overlap (or enrichment) was statistically significant by permutation test (p<10^−4^ by 1,000,000 permutations). Specifically, the number of genes that overlap between the cortex and SPC genes was significantly greater than that of random gene sets of the same sizes (190 as cortex genes and 259 as SPC genes) drawn independently from the total gene pool from which the cortex and SPC genes were identified. Ten out of 132 transcripts highly expressed in the glomerulus were found in the SPC gene list, representing a significant overlap between the two gene lists although to a lesser degree than the cortex genes (p = 0.005 by 1,000,000 permutations). The glomeruli are located exclusively in the normal renal cortex and genes expressed in isolated glomeruli reflect additional expression features of differentiated normal renal cortex. No significant overlap was found between SPC gene set and gene lists from other anatomic segments of the kidney. Specifically, 2 of 425 transcripts highly expressed in the renal pelvis were found in SPC gene list and none of the transcripts highly expressed in the medulla or papillary tips were found in the SPC gene list. All individual gene lists and overlapping genes are provided in Supplemental file S1.

We also compared the SPC gene set with the CSR gene set, a gene expression program that captures the response of fibroblasts to serum exposure that is thought to reflect the stereotypical transcriptional programs activated in fibroblasts during wound healing [9]. Eighteen out of 353 unique named core serum response genes were found in the SPC gene set, representing a significant enrichment (*p*<10^−5^ by Chi-square test). All 18 genes were repressed by serum exposure in fibroblasts, but showed variation in expression levels across the ccRCC samples. Taken together, the normal cortex, glomerulus, and CSR gene sets captured more than 20% of the genes in the highly prognostic SPC gene set. This suggested the hypothesis that loss of normal cortical differentiation and gain of features associated with wound healing are important biological features associated with lethal ccRCC.

### Expression of normal renal cortex and glomerulus genes predict survival in ccRCC

Out of the 190 named unique genes that are predominantly expressed in the renal cortex, 67 genes represented by 87 transcripts were well measured and highly variable in the ccRCC data set (for a complete list of genes and expression levels, see Supplemental file S2). To test the hypothesis that loss of gene expression features of the normal renal cortex predicts survival of ccRCC patients, we first used hierarchical clustering to sort the 177 ccRCC samples based on the 87 “normal cortex” transcripts found in the ccRCC dataset. As shown in Figure 1A, average linkage clustering partitioned the samples into two main groups with predominantly high or low levels of expression of the normal cortex transcripts (a detailed view of the sample cluster dendrogram is available in supplemental Figure S1 and the patient survival information in Supplemental file S3). Kaplan-Meier survival analysis showed that the patients whose tumors most resembled normal cortex with high levels of normal cortex gene expression (cluster 1) had a higher probability of cancer-specific (Figure 1B, *p* = 0.00007, log-rank test) and overall survival (Figure 1C, *p* = 0.0003, log-rank test) compared to those with low expression of cortex genes in their tumors (cluster 2). The median overall survival for patients in cluster 1 was 39.5 months compared to patients in cluster 2 which was 22 months. The median disease specific survival for cluster 1 was 41 months and cluster 2 was 21 months.

In addition, we used alternative methods to validate the predictive power of cortex genes. First, we calculated the scaled gene expression score by summing up the log2 based gene expression ratios of the 87 cortex transcripts well measured in the 177 cRCCs while taking into account the direction of the gene expression. The distributions of the scores for cluster 1 and cluster 2 were shown in the histogram below the heat map in Figure 1. Based on the distributions, the sums of the log2 ratios for the cortex transcripts were overexpressed in the majority of cases in cluster 1 with good prognosis compared to cluster 2 with poor prognosis. Second, we divided the samples into two groups at the median of the sum scaled gene expression and used the group label for survival analysis. The group with high expression of cortex genes had a significant better survival than the group with low expression (p = 0.00246, likelihood ratio test), confirming the predictive power of the cortex gene set.

Like the cortex gene list, the glomerulus genes are all expressed at high levels in isolated glomeruli from the normal renal cortex, therefore, high expression of these genes in ccRCC samples reflects similarity to normal glomerular gene expression patterns. Fifty out of the 132 transcripts highly expressed in glomerulus were well measured and highly variable in the ccRCC data set. We calculated the scaled gene expression by summing up the log2 based gene expression ratios of the 50 glomerulus transcripts while taking into account the direction of the gene expression. In a univariate model, high expression for the glomerulus genes predicted improved survival (p = 0.0003). Thus, a gene set that is enriched for a cell population of the normal cortex also predicted outcome in ccRCC. Because of the expected overlap in the patterns of gene expression in the glomerulus gene set with the normal cortex, we focused our analysis on the cortex gene set.

A Cox proportional hazards model was used to evaluate the significance of the “normal cortex” gene expression signature in the context of known clinical prognosticators. Tumors were sorted into two groups (those whose expression patterns more closely resembled normal cortex and those that did not) based on the above hierarchical cluster analysis using the cortex gene set. When used as a categorical variable, the cortex gene set was a strong predictor of survival, independent of tumor grade, tumor stage and patient performance status (Table 1). We also evaluated expression across the cortex gene set as a continuous variable. For each tumor sample, we computed a score that represented the sum of the normalized expression levels for each of the 87 transcripts from the cortex gene set. Since all transcripts in the cortex gene set are expressed at high levels in the normal renal cortex, tumors with high scores had expression patterns that most resembled the normal renal cortex. When the cortex gene expression score was used as a continuous variable, it better predicted outcome than the categorical analysis. Tumors with high cortex gene set scores showed significantly improved survival, independent of the clinical prognostic factors (Table 1). Intriguingly, the glomerulus gene set also predicted outcome as a continuous variable independent of grade, stage and performance status (p = 0.004), although to a lesser degree than the cortex gene set (p = 0.0005).

**Table 1:** Multivariate analysis of prognostic values of cortex gene signature and other clinical parameters.

Variable	p-value*	Variable	p-value*
Cortex-categorical	0.0059	Cortex-continuous	0.0005
Grade	0.4499	Grade	0.302
Performance status	0.0021	Performance status	0.035
Stage	<10^−4^	Stage	<10^−4^

* p-values from the analysis of deviance (likelihood ratio test).

### The SPC genes are highly expressed in the normal renal cortex

**Figure 2:**
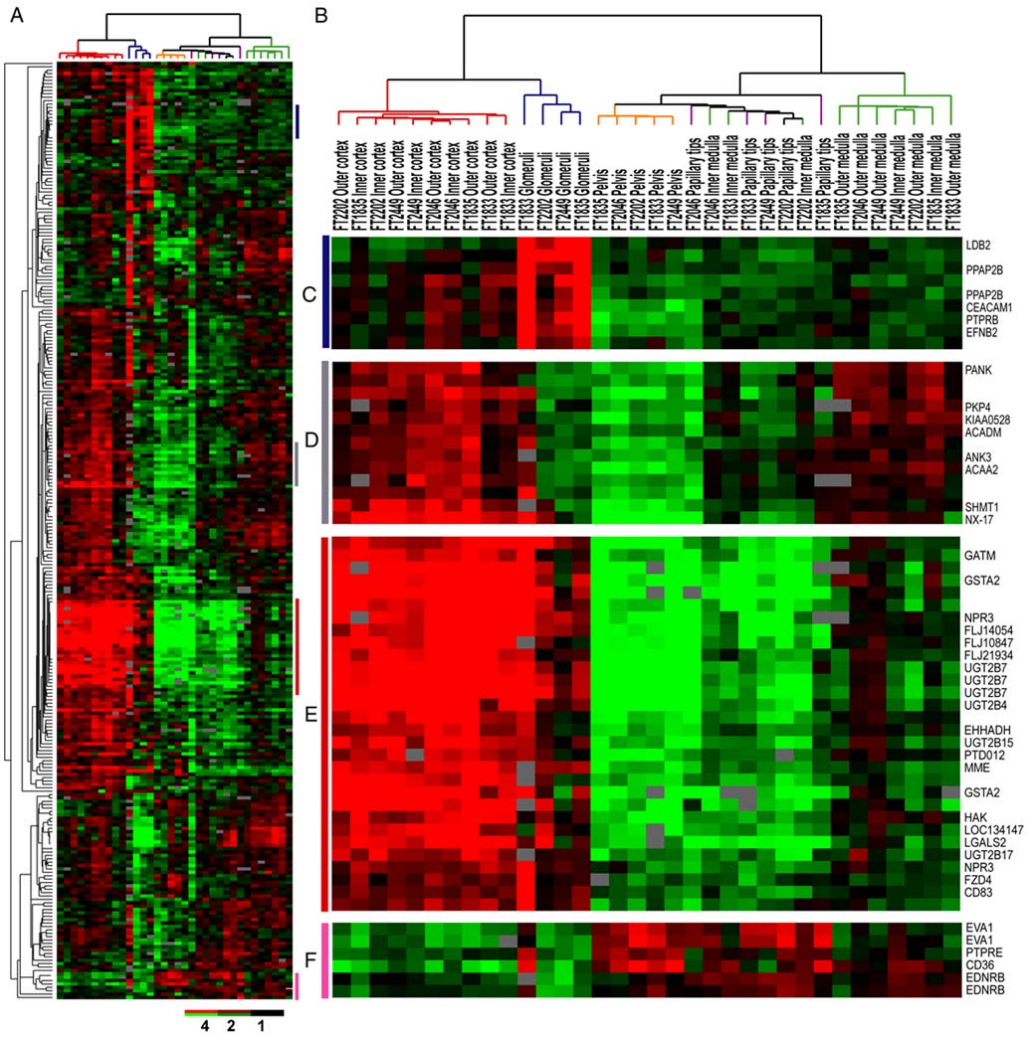
Hierarchical Clustering Analysis of 34 normal kidney samples using 259 SPC genes. (A) Overview of the gene expression patterns of SPC genes across the 34 samples. Colored bars identify the locations of the enlargements of the expression map in (C–F). (B) Cluster dendrogram representing similarities in the expression patterns between experimental samples. Samples were separated into two main groups and five subgroups (cortex in red, glomeruli in blue, pelvis in orange, medulla in green, and papillary tips in purple). (C) SPC genes specifically overexpressed in glomeruli. (D) SPC genes specifically overexpressed in cortex and medulla. (E) SPC genes specifically overexpressed in cortex. (F) SPC genes specifically underexpressed in cortex and glomeruli.

Given the overlap between the cortex and SPC gene sets, we were curious about the extent to which the SPC gene set captured normal renal cortical differentiation. We used the SPC gene set to perform hierarchical clustering analysis of 34 normal kidney samples that had been dissected from different regions of 5 adult kidneys [8]. Samples from distinct anatomical regions of the kidney were segregated with few errors based on their expression patterns across the SPC gene set (Figure 2). Samples from the cortex and purified glomeruli were clearly separated from the samples from the medulla and pelvis. Furthermore, the glomerular samples formed a distinct group from the cortical samples, while 7 of the 10 the medullary samples clustered separately from the pelvis and the papillary tips. Samples of pelvis clustered on a branch together with all the papillary tips. Three of the 5 inner medulla samples cluster with papillary tips, while the other two clustered with the outer medulla samples. The majority of the SPC genes showed high expression in cortex samples. Most notable included a number of detoxification enzymes such as several UDP glycosyltransferase 2 family members (UGT2B4, 7, 15, and 17) and Glutathione S-transferase A2. Therefore, the SPC gene list captured gene expression features of normal renal differentiation and best classified the normal cortex samples compared to the other anatomical segments of the kidney.

### Expression of CSR genes predicts survival in ccRCC

**Figure 3:**
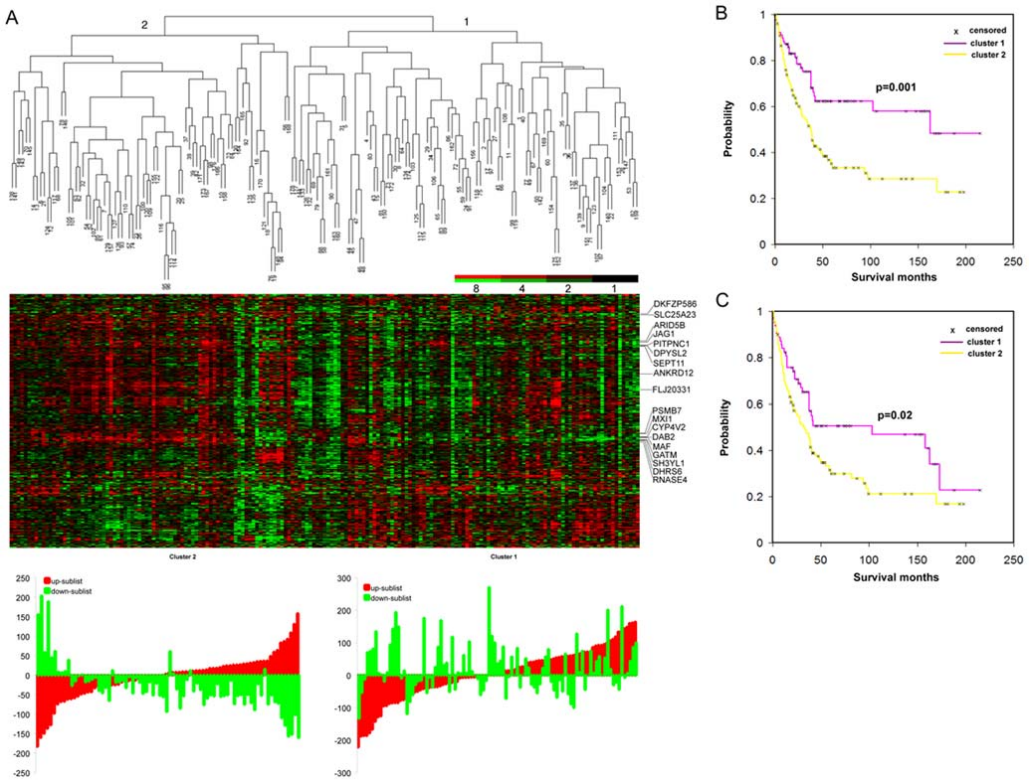
Hierarchical clustering based on core serum response (CSR) gene expression predicts survival in ccRCC. (A) Supervised hierarchical clustering analysis of the expression patterns of 353 CSR genes represented by 420 transcripts in 177 ccRCCs. The distributions of the scaled gene expression scores for the up- and down-sublists were shown below the heat map. (B) Kaplan-Meier estimates of disease-specific survival of two main groups of ccRCC patients defined in (A) based on the CSR gene expression. (C) Kaplan-Meier estimates of overall survival of two main groups of ccRCC patients defined in (A) based on the CSR gene expression.

The transcriptional signature of the response of fibroblasts to serum has been shown to be a powerful predictor of the clinical course in several common carcinomas [9], [11]. The enrichment of CSR genes in SPC gene set suggests that CSR signature may predict survival in ccRCC and might reflect biological features captured in the SPC gene list. Out of the 400 unique genes in the core serum response signature, 353 represented by 420 transcripts were well measured and highly variable in the ccRCC data set (for a complete list of genes and expression level, see Supplemental file S4). Out of the 420 transcripts, 207 were downregulated (down-sublist) by serum treatment and 213 were upregulated (up-sublist). When average linkage hierarchical clustering analysis of the 177 patient samples was performed using this 353 genes, tumors were partitioned into two main groups (Figure 3A and Supplemental Figure S1). We calculated the sum of the expression ratios for the up- and down-sublist for each tumor, and the distributions of the sum for cluster 1 and cluster 2 were shown in Figure 3A. The majority of cluster 2 tumors showed high expression of the up-sublist and low expression of the down-sublist, suggesting an activation of serum response program. In addition, more cluster 1 tumors than cluster 2 tumors showed low expression of the up-sublist and high expression of the down-sublist, indicating an inversed expression of serum response program. Finally, in the cluster 1 tumors there was a small subset of tumors in which the up-sublist and many of the down-sublist of genes were expressed at a higher level, suggesting that the up- and down-sublists were not coordinately regulated in these tumors. Kaplan-Meier analysis showed that cluster 1 had higher cancer-specific (Figure 3B, *p* = 0.001, log-rank test) and overall survival (Figure 3C, *p* = 0.02, log-rank test) compared to cluster 2. The median overall survival for cluster 1 was 38 months and for cluster 2 was 25.5 months. The median disease specific survival for cluster 1 was 38 months and for cluster 2 was 27 months. These results suggest that activation of the CSR gene expression program predicts ccRCC survival.

**Figure 4:**
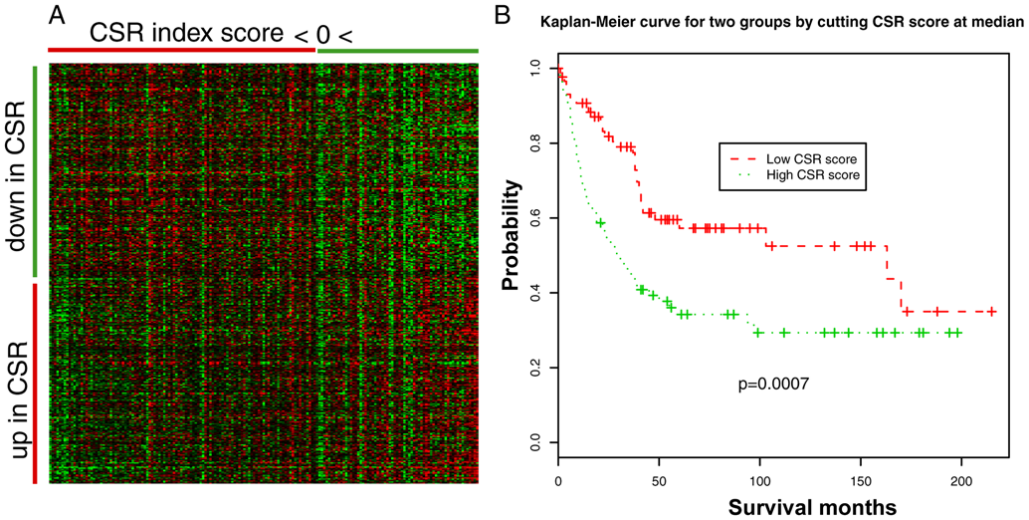
Weighted scaling expression of CSR genes distinguished ccRCC patients with better survival vs. poor survival. (A) Heat map of CSR gene expression in 177 ccRCC patients. CSR genes were separated into up- and down-sublists based on their response to serum. ccRCC samples were sorted by the sum of CSR gene expression ratios in each tumor calculated while taking into account their direction of changes in response to serum. (B) Kaplan-Meier estimates of disease-specific survival of two groups of ccRCC patients defined using the median of the weighted scaling expression of CSR genes as a cutoff.

We further examined the predictive power of CSR genes using an alternative weighted scaling method. Specifically, a CSR index score was calculated for each sample by summing up the expression ratios taking into account the direction of regulation of each gene by serum treatment (see Methods). We sorted the tumors by their CSR index score and grouped the genes by their direction of change in response to serum treatment (Figure 4A). Tumors with high CSR scores were those that displayed gene expression similar to wound healing (i.e. high expression of genes upregulated by serum stimulation and low expression of genes downregulated by serum stimulation). We separated the 177 tumors into two groups using the median CSR index score as the cutoff. Kaplan-Meier analysis using this grouping criterion showed tumors with high CSR scores had significant lower cancer-specific survival than those with low CSR scores (p = 0.0007) (Figure 4B). These results confirmed that activation of the wound-healing gene expression signature is associated with poor survival in ccRCC patients.

Multivariate Cox proportional hazards analysis was used to evaluate the CSR gene list and patient grade, stage, and performance status in predicting outcome. Tumors grouped by hierarchical clustering with greater resemblance to the CSR showed significantly poorer survival compared to those that showed little CSR expression signature, independent of the clinical variables. Used as a continuous variable, the weighted CSR score (based on a sum of the sign-corrected 420 CSR transcripts) better predicted of cancer specific survival independent of tumor stage and grade and patient performance status (Table 2).

**Table 2:** Multivariate analysis of prognostic values of CSR gene signature and other clinical parameters.

Variable	p-value*	Variable	p-value*
CSR-categorical	0.0206	CSR-continuous	0.0473
Grade	0.475	Grade	0.2062
Performance status	0.0030	Performance status	0.0037
Stage	<10^−4^	Stage	<10^−4^

* p-values from the analysis of deviance (likelihood ratio test).

### Robustness of survival prediction by cortex and CSR genes

We compared random gene lists of the same size as the cortex and CSR gene lists to test the robustness of survival prediction by these two gene sets. For cortex gene list, 1,000,000 random transcript lists with 87 transcripts were constructed and the sum of expression levels across each gene list was used to predict survival in a univariate Cox model and the p-value was recorded. None of the 1,000,000 p-values was smaller than that from the cortex gene list, suggesting a <10^−6^ p-value for the significance of cortex gene list.

For the CSR gene list, the same simulation was performed and the sign information was also randomly distributed on each simulated gene list. The signed sum was used to predict survival. Among the 1,000,000 p-values recorded, 3193 records were smaller than that from the CSR gene list, which suggested a p-value of 0.0032 for the significance of CSR gene list.

### Relationships between the Cortex, CSR, and SPC gene sets

The normal renal cortex and CSR signatures overlapped significantly in their classification of the 177 ccRCCs into good or poor prognostic groups. Based on group assignment from the hierarchical cluster analysis, both the CSR and normal cortex gene sets categorized 53 cases as having favorable prognosis and 67 as having poor prognosis. As shown in Table 3, approximately 70% of the tumor samples in hierarchical cluster groups with similar outcomes overlapped (i.e. cluster 1 in Figure 1 and cluster 1 in Figure 3). The similarity in prognostic group assignment was highly significant (*p*<10^−5^ by Chi-square test). Moreover, the median cortex gene expression score calculated by weighted scaling method for the favorable group was 32.02 while it was −29.74 for the group with poor survival. On the other hand, the median CSR score calculated similarly was −73.80 in the favorable group and 23.48 in those with poor survival. The cortex score and CSR score were inversely correlated (R = −0.5029), implying that tumors with favorable outcome more closely resembled the cortex signature and less resembled the core serum response signature. These results demonstrated that the majority of the tumors simultaneously showed higher degree of cortical differentiation and lower level of wounding healing or lower level of cortical differentiation and higher level of wound healing. In other words, gene signatures associated with prognosis were concurrently expressed in the majority of ccRCCs. In multivariate analysis, the SPC gene set did not predict outcome independent of the normal cortex gene set. Furthermore, the cortex, glomerulus and CSR gene sets did not predicted survival independent of the SPC gene set, implying that SPC gene set captures essential features of the cortex, glomerulus and CSR gene sets.

**Table 3:** Comparison of the groupings using the cortex and the CSR genes.*

	Cluster 1 (Figure 3)	Cluster 2 (Figure 3)	Total cases	Outcome
**Cluster 1 (** **Figure 1** **)**	53	31	84	good
**Cluster 2 (** **Figure 1** **)**	26	67	93	bad
**Total cases**	79	98		
**Outcome**	good	bad		

* The overlapping is statistically significant by Chi-square test (p<10^−5^).

## Discussion

Clear cell RCC appears to arise from cells in the proximal convoluted tubules of the renal cortex [13], [14]. Inactivation of the VHL gene and consequent activation of the HIF1 hypoxic signaling pathway are nearly universal features of ccRCC and appear to be critical to ccRCC carcinogenesis [15]. More mysterious are the features of ccRCC biology that drive aggressive disease and underlie lethality. Perhaps the most striking feature of prognostic gene sets arising from profiling studies of Takahashi and colleagues and our group is that nearly all transcripts correlated with survival show lower levels of expression in the most aggressive tumors. We observed highly significant overlap between our prognostic (SPC) gene list and a set of genes highly expressed in the normal renal cortex and normal glomeruli. Loss of expression of the normal cortex and glomeruli genes in the ccRCCs was associated with decreased survival, and the prognostic value of the normal cortex genes was similar to (and not independent of) the SPC gene list. The SPC prognostic gene list was able to correctly sort samples from anatomical segments of the normal kidney and was particularly good at grouping samples from the normal cortex and glomeruli. Therefore, one of the most important biological features of the SPC gene set is that it captures the transcriptional program of the terminally differentiated normal renal cortex. Loss of the transcriptional program associated with renal cortical differentiation is an important feature of aggressive ccRCC and highly predictive of cancer-specific death.

The SPC gene set also appears to embody features of the core serum response. Again, there was significant enrichment for the CSR genes in the SPC prognostic gene set. The CSR set also was highly predictive of survival in ccRCC, independent of known clinical prognostic parameters. Intriguingly, tumors with expression features that more closely resemble the CSR gene set show less similarity to the expression patterns of the normal renal cortex. In other words, loss of transcriptional patterns associated with normal cortical differentiation was associated with acquisition of those of the core serum response. The CSR signature does not reflect proliferation since all of the proliferation-related genes have been removed by cross-referencing to a comprehensive list of cell-cycle regulated genes identified by Whitfield et al. [16]. The core serum response does display many aspects of wound healing and previous work has suggested that the cancer microenvironment resembles a wound that does not heal [17]. Similarities in gene expression profiles of ccRCC and renal regeneration and repair have been reported previously, although this relationship was not tested for effects on clinical outcome or survival [18]. The CSR has been found to provide independent survival prediction in several of caner types [9], [11] and our study is the first to show that it predicts survival in ccRCC.

The absence of the CSR signature in the ccRCCs with favorable outcomes might reflect a tumor microenvironment that does not promote tumor progression. Alternatively, since fibroblasts invoke features of mesenchyme during wound healing including activation and migration, it is conceivable that acquisition of “wound healing” is associated with EMT. In addition, loss of differentiation and gain of EMT during tumor progression has been observed in a number of tumor types [19]. For example, gastric cancer cells with fibroblastoid morphological changes show increased motility and invasiveness due to decreased cell-cell adhesion, reminiscent of EMT during embryonic development [19]. EMT has been reported to be an integral component of colorectal and breast cancer progression [21], [22]. In our dataset, concomitant loss of differentiation and acquisition of “wound healing” was associated with features of EMT including down regulation of the epithelial marker CDH1 and up regulation of the mesenchymal markers FN1 and VIM (for detailed information see Supplemental file S5 and S6), suggesting that EMT may play a role in clear cell renal cell carcinoma progression.

The use of normal tissue gene expression signatures for prognostication has been reported in diffuse large cell B lymphomas (DLBCL) [23]. Alizadeh and co-workers identified two molecularly distinct forms of DLBCL that have gene expression patterns resembling different stages of B-cell differentiation. DLBCLs that express genes characteristic of normal germinal center B cells have significantly better overall survival than those with expression features resembling circulating blood B cells that have been activated in vitro. Taken together with our data, these findings suggest that better understanding of normal tissues and the programs that regulate their development could provide insights into cancer progression. Furthermore, our findings suggest that expression patterns from normal tissues have potential as prognostic signatures for the malignancies that arise from them. Similarities in gene expression patterns between tumors with favorable prognosis and normal tissues also strengthens the rationale of promoting normal differentiation in malignant cells as an alternative to cytotoxic therapy or as a secondary prevention strategy.

## Supporting Information

File S1 Comparison of SPC genes with normal kidney gene sets
(0.37 MB XLS)

File S2 Expression levels of cortex genes in 177 ccRCC patients
(0.30 MB XLS)

File S3 Patient ID and survival information
(0.04 MB XLS)

File S4 Expression levels of CSR genes in 177 ccRCC patients
(1.40 MB XLS)

File S5 Expression levels of EMT markers in 177 ccRCC patients
(0.03 MB XLS)

File S6 Expression of epithelial-mesenchymal transition (EMT) markers in 177 ccRCC patients.
(0.03 MB DOC)

Figure S1 Large-size dendrograms in Figure 1 and 3
(5.77 MB TIF)
