# Supplementary material for: Alteration of Gene Expression Signatures of Cortical Differentiation and Wound Response in Lethal Clear Cell Renal Cell Carcinomas
Source: PLoS One. 2009 Jun 25;4(6):e6039. doi: 10.1371/journal.pone.0006039 (PMC2698218; doi:10.1371/journal.pone.0006039)
Supplement: File S6 — Expression of epithelial-mesenchymal transition (EMT) markers in 177 ccRCC patients. (0.03 MB DOC) [file pone.0006039.s006.doc]

***Expression of epithelial-mesenchymal transition (EMT) markers in ccRCC***

Since wound healing and EMT share similar features and EMT has been implicated in tumor progression, we examined the expression of four well-known EMT markers [12], namely E-cadherin (CDH1), N-cadherin (CDH2), fibronectin (FN1) and vimentin (VIM) in our dataset. CDH1 is an epithelial cell marker while CDH2, FN1, and VIM are mesenchymal markers. Consistent with the fact that CDH1 is downregulated while FN1 and VIM are upregulated during EMT, the expression of CDH1in our data set was inversely correlated with FN1 and VIM with Pearson correlation coefficient of -0.39 and -0.25, respectively. Expression of CDH2, which is also upregulated during EMT, was positively associated with that of FN1 or VIM in our data set; Pearson correlation coefficient was 0.20 and 0.38, respectively. These results demonstrated that these EMT markers showed coordinated expression in ccRCC patients.

Using expression levels of these four markers (see Supplemental file 5), we stratified the 177 ccRCCs into a high EMT group and a low EMT group by hierarchical clustering. The overlapping between EMT and cortex or CSR clusters was shown in Table S1. The enrichment of high EMT tumors in low cortex and high CSR groups was statistically significant (p<10^-2^ and 10^-6^ by chi-square test, respectively), suggesting that loss of differentiation and gain of wound healing is associated with EMT in ccRCC patients.

Table S1 Comparison of the groupings using the cortex, CSR, and EMT genes*

|  | **Low EMT** | **High EMT** | **Total cases** | **Outcome** |
| --- | --- | --- | --- | --- |
| **Cluster 1 (Figure 1 cortex)** | 56 | 28 | 84 | good |
| **Cluster 2 (Figure 1 cortex)** | 40 | 53 | 93 | bad |
| **Cluster 1 (Figure 3 CSR)** | 59 | 20 | 79 | good |
| **Cluster 2 (Figure 3 CSR)** | 37 | 61 | 98 | bad |
| **Total cases** | 96 | 81 |  |  |
| **Outcome** | good | bad |  |  |

* The overlapping between EMT and cortex or CSR is statistically significant by Chi-square test (p<10^-2^ and 10^-6^, respectively).
