# Supplementary figures and images for: Alteration of Gene Expression Signatures of Cortical Differentiation and Wound Response in Lethal Clear Cell Renal Cell Carcinomas
Source: PLoS One. 2009 Jun 25;4(6):e6039. doi: 10.1371/journal.pone.0006039 (PMC2698218; doi:10.1371/journal.pone.0006039)

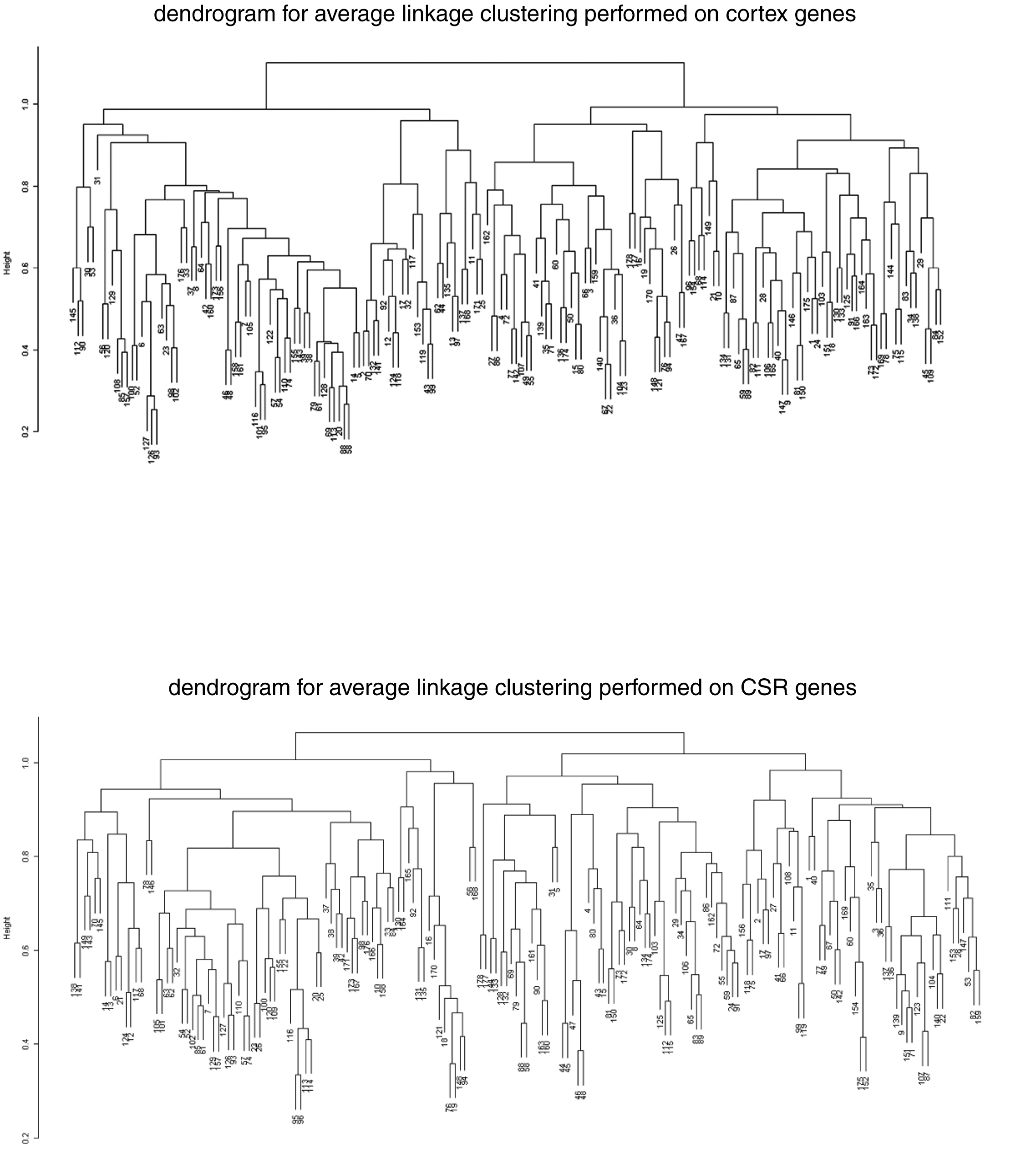

Supplement: Figure S1 — Large-size dendrograms in Figure 1 and 3 (5.77 MB TIF) [file pone.0006039.s007.tif]
